# Walkability assessment of metro catchment area: A machine learning method based on the fusion of subject-objective perspectives

**DOI:** 10.3389/fpubh.2022.1086277

**Published:** 2022-12-06

**Authors:** Peng Zeng, Weixing Xu, Beibei Liu, Yuanyuan Guo, Linfeng Shi, Meng Xing

**Affiliations:** ^1^School of Architecture, Tianjin University, Tianjin, China; ^2^School of Architecture & Fine Art, Dalian University of Technology, Dalian, China; ^3^College of Intelligence and Computing, Tianjin University, Tianjin, China

**Keywords:** metro catchment area, walkability, built environment, machine learning, efficient imbalance-type factors, optimization strategies

## Abstract

China's metro system is developing rapidly. Walking is the most frequently adopted mode to connect to the metro, the attributes of the pedestrian-built environment around the stations directly influence people's willingness to use the metro. However, few studies have paid attention to the comprehensive assessments of the built environment in the metro catchment area. Thus, this paper attempts to construct a walkability evaluation model that combines subjective and objective perspectives. We collected field data of the built environment factors affecting on walkability in the 800 m buffer zone of eight case metro stations in Dalian city, China. We also collected on-site interviews from 867 passengers to evaluate the walkability. A machine learning-based approach was developed to calculate the weights of walkability variables, followed by constructing a *Score-Effectiveness* framework to identify the built environment factors in the metro catchment area that need to be improved. The study found that the shading facilities, obstacle barriers, and resting seats around pedestrian walkways are the most efficient and imbalanced variables recognized by the crowd. The convenience of overpasses and underpasses are additional efficient imbalance-type variables for leisure and commuting populations, respectively. This indicates that the current level of construction of the above five built environment factors is relatively low, but the construction has a significant impact on the degree of friendliness in supporting pedestrian walkability. In this paper, improvement measures are proposed in a targeted manner in order to achieve the effect of effectively improving the current level of metro catchment area's walkability. The results of the study can provide references to provide strategies for precise pedestrian planning in the metro catchment area, leading to a pedestrian environment with high walking quality.

## Introduction

The metro is featured with safety and punctuality, leading to an advantage of avoiding congestion caused by uncertainty compared to other modes of ground transportation. Thus, an increasing number of travelers choose the metro as their primary mode of transportation. The popularity of the metro has led to a rapid increase in pedestrian flow within the catchment area, which in turn has triggered changes in land values and has had a positive impact on housing prices around the stations (1). Even the impact of the COVID-19 epidemic does not jeopardize the price trend of real estate, indicating that the metro has a strong appeal to people (2). At the same time, the metro itself and its combination with other modes of transportation can significantly reduce urban carbon emissions, improve air quality, reduce noise caused by traffic, and promote the physical activity of travelers (3–6). In the context of calling for carbon neutrality in various countries around the world, metro transportation is a strong pillar of sustainable transportation systems. In light of this, effectively guiding travelers' shift from individual motorized transport to transit transportation is the major challenge for sustainable urban transport development in the future faced by planners and policymakers (7–9). By the end of 2021, 48 cities in mainland China have opened metros, with an operational mileage of 7209.7 km, ranking first in the world and showing an increase of 14.8% compared to 2019. The increasing number indicates that China is currently in a period of rapid development of metro transit, and the metro is becoming more and more prominent in China's urban transportation system.

As the most frequently used mode of transportation, walking usually possesses a higher level of satisfaction compared to public transportation and automobiles (10). Improving the pedestrian environment around metro stations will encourage people in the vicinity of the station to walk to the station instead of using other transport modes (11), but travelers are also limited by the time spent and the distance traveled within a certain range (12, 13). Yet, when the purpose of the trip is clear, travelers may also undertake more walking distance and walking time (14). In this sense, the improvement of the walking environment in the metro catchment can fit with the primary goal of Transit Oriented Development (TOD), which aims to create pedestrian-friendly and livable communities that are closely linked to public transit stations (15). Related results highlight that the pedestrian friendliness of the built environment around a site affects people's choice of travel mode, and improving walkability will significantly increase the likelihood that travelers will choose to walk to the site (16). Nevertheless, how the subjective and objective built environment factors of the current situation affect the walkability of the metro catchment areas these factors have not been fully explored (17).

Furthermore, by taking eight metro stations in Dalian, China as a case study, this study attempts to establish a comprehensive framework for the evaluation of the walking environment of the metro catchment area. The framework combines the subjective environmental perception of pedestrians and the objective environmental accessibility of the metro catchment area. In the framework, a machine learning-based approach is developed to systematically identify the weight of key factors that affect the walkability of metro catchment areas. It is expected that the development framework can provide policymakers and planners with references to form strategy directions for improving the walkability environment of metro catchment areas.

The remainder of this paper is organized as follows. Section Literature review presents the review work on the concept of walkability, followed by an overview of influencing factors of walkability, methods of walkability evaluation, and walkability in the metro catchment areas; Section Data introduces the study area, data collection, and description; Section Methodology describes the idea of evaluating the walkability of metro catchment areas, including the introduction of a new method of machine learning; Section Results explains the statistical and modeling results, followed by Section Discussion which holds the discussion and offers policy and practice implications; Section Conclusion concludes the paper and summarizes research limitations.

## Literature review

### Walkability and influencing factors

*Walkability* refers to the ability of humans to walk (18). In previous studies, several scholars and experts have proposed different definitions of *walkability* from various research perspectives. In the field of urban planning, *walkability* means the degree to the features of the built environment that are friendly to residents' living, commuting, shopping, and leisure (19). In other words, it means how pedestrian-friendly the urban space is (20), and thus, walkability is frequently used to assess the friendliness of walking in an area (21) and then to promote the active behavior of walking (22). Walkability is also considered to be one of the basic criteria of urban planning to counteract urban sprawl by creating good walkable areas for different activities and services (23). In the field of public health, friendly walkable areas can serve as a mental incentive for people to actively take up walking, which can effectively prevent cardiovascular diseases and enhance physical and mental health (24). In addition, scholars in geography have used detailed traffic data to understand the relationship between the built environment and walking behavior (25). By reviewing previous related studies, walkability in this study is defined as the degree to which the built environment allows walking (26) and encourages people to walk in a friendly manner (20, 22). Usually, areas with high walkability can encourage people to increase their subconscious maximum walking distance (27). Thus, improving pedestrian infrastructure in the metro catchment area can strengthen the willingness of travelers to connect to the metro by walking and effectively increase ridership (28).

The built environment factors that influence walkability are multifaceted and can be broadly divided into mesoscale neighborhood factors and microscale pedestrian factors. The former are mainly presented at the neighborhood or community level, such as street connectivity, residential density, and the mix of land uses associated with daily use facilities (29–33). The latter places more emphasis on walkability, focusing on the real pedestrian experience, such as street furniture, walkway width and quality, and other micro factors that affect pedestrian perception and experience (34, 35).

Although most studies have used mesoscale walkability as a research lens, few scholars hold to argue that built environment factors, such as neighborhood size, street network density, and community-level facility diversity, do not reflect how pedestrian-friendly facilities are (36). Also, factors that present positive effects at the mesoscale may even become negative at the microscale, such as road intersection density. For instance, a higher road intersection density represents better connectivity (31, 37). On the contrary, at the microscopic level, the increase in the density of roadway intersections may cause safety concerns due to frequent street crossings (35). As a result, increasing attention has been paid to walkability studies at the microscopic scale, which can truly depict the conditions of the pedestrian-built environment (36). Recent studies on the built environment affecting walkability have focused on walking paths, sidewalks, and lighting at the microscopic scale (32, 35). Walkable areas should also be enhanced with urban imagery such as historic buildings and public artwork, all of which can improve the quality of the pedestrian environment (38). As such, the walkability of an area is largely influenced by the built environment, such as pedestrian trail facilities and pedestrian services (39).

### Walkability evaluation

Evaluating how pedestrian friendly an area is can be divided into two perspectives: a subjective or an objective evaluation. People living in the same built environment may exhibit different willingness to travel due to their individual judgments on the walkability of potential routes (40, 41). As such, the spatial experience of the walking process (42) affects the characteristics of their trips (43).

Subjective methods to evaluate walkability are usually based on questionnaires to obtain pedestrians' subjective perceptions of the built environment, such as the Neighborhood Environment Walkability Scale (NEWS), Semantic Differential (SD), Public Life in Public Space (PLPS), Neighborhood Quality of Life Study (NQLS) and Pedestrian Environment Review System (PERS) (44–46). Among them, the NEWS is a widely adopted method for subjective evaluation on walkability (47). The assessment variables involve with several dimensions such as comfort, safety, and convenience (43). The assessment of the quality of the street environment captures people's perceptions of the pedestrian environment (48), including pedestrians' opinions on the aesthetics of the surrounding built environment, pedestrian traffic safety, social crime safety, or neighborhood satisfaction (49).

Studies evaluating walkability by objective methods typically construct multiple environmental variables (50), which are then integrated and calculated to obtain a score value to measure walkability (51). Various types of examples, walkability index, which helps to quantitatively measure walkability, have been proposed by many scholars (52, 53). Particularly, Walk Score is the most popular and commonly used quantitative tool for objectively calculating walkability degree. The tool was introduced in 2007 by a US company to promote walkable communities and has become popular among real estate agents to promote walkable urban areas. The Walk Score considers the vicinity of different facilities *via* walking by taking into account the attenuation effects due to walking distance, intersection density, and block length (54). However, some scholars point out that the definition, classification, and importance of facilities as well as the raster-based distance decay pattern may differ among different urban regions. This also leads to the problem of non-comparability of walk scores in different regions, especially in high-density Asian regions that differ significantly from North America (28). For example, Hino et al. developed the Japanese Walkability Index (JWI) to measure walkability in cities based on the high-density urban structure of Yokohama. Since the JWI preferentially considers amenities, road networks, and land prices rather than density variables, it is superior for high-density Asian cities (55). Moreover, the European urban context is distinctive from that of US, which featured with low population density, low land use mix, and connectivity. To obtain a walkability index more in line with the European urban context, European scholars adjusted the choice of environmental variables and the size of weights in the walkability index. For example, Grasser et al. (25) used population density, household density, land use mix entropy index, and three-way cross density to construct a walkability index that fits the European regional context.

Moreover, it has been shown that Walk Score presents a positive correlation with both objective built environment factors (29) and the subjective perceived built environment factors (56–60). This suggests that the Walk Score should consider the effect of individual's perception, rather than only deriving from objective evaluation. Actually, the pedestrian experience during walking also plays a corresponding role in promoting or inhibiting walking, such as the degree of sidewalk continuity, safety during walking (61), and sidewalk quality (35).

### Walkability of the metro catchment area

The concept of walkability is used by Dutch scholars to explain the link between the built environment and walking as an important component of TOD (37). Current policies also tend to promote a shift to non-motorized and public transportation (62, 63). However, improving the efficiency of public transportation alone is not enough to improve the ridership of public transportation. Enhancing the availability of public transportation (i.e., metro transit) *via* walking is also essential for promoting the public transport usage. Obviously, inadequate walkability around transit stations may reduce the patronage of the metro (28, 64). For instance, scholars in Italy have shown that public transportation in the country is currently inefficient and unreliable because the built environment in the vicinity of a station is not friendly enough for people to walk, including factors such as insecurity for pedestrian safety, low-quality sidewalks, and impassability due to the presence of obstacles on the sidewalk (35). In addition, the level of walkability in the metro catchment area is significantly and positively correlated with the probability of choosing to walk to and from the station. The study on metro passenger transfer modes by Wu et al. (65) showed that the quality of the pedestrian environment in the metro catchment area was positively related to the probability of passengers choosing walking as a transfer mode. In a medium-sized urban context in Colombia, Arellana et al. (66) also argue that the walkability and friendliness of the built environment, such as sidewalk condition and attractiveness, play an important role in pedestrian travel mode decisions.

Although poor walkability has a debilitating effect on people's willingness to use the metro, the adoption of walkable connections to stations has not yet received enough attention (67). Factors affecting metro catchment area walkability can be divided into mesoscale and microscale. Most previous studies on walkability have focused on built environment factors at the mesoscale, including land use diversity (68), density (69, 70), street design (71), connectivity (72), distance, and accessibility within the metro catchment (73). However, most of the studies are deficient in assessing pedestrian perceptibility of the walking environment at the microscopic scale. Microscopic factors of the built environment have an important influence on the walkability around metro stations (73), which can either facilitate or inhibit pedestrians (74). Moreover, the level of walkability also correlates to an individual's satisfaction with the perceived built environment (75, 76), including the quality and continuity of sidewalks (28), lighting (77), and the degree of obstruction on the walkway (78). Additionally, factors that are essentially subjective in nature, such as the degree of sidewalk cleanliness, social security around the station, traffic safety, and signage, are also found to have an impact on the walkability of the metro catchment area (79, 80).

In summary, although previous methods of evaluating walkability have involved objective spatial factors and subjective psychological factors, few studies have yet been conducted to combine subjective and objective evaluation variables. Meanwhile, the traditional methods of weight determination include correlation analysis and regression analysis, but they involve different subjective and objective dimensions. Further, such methods of pre-determined functional relationships may bring bias in the results. In addition, previous studies have not adequately combined the current built environment of the metro catchment areas with its importance to walkability, which is not conducive to accurately exploring the current shortcomings of the metro catchment area's walkability.

## Data

### Study area

Dalian, a tourist-oriented city, has a population of about 598.7 million and achieved a GDP of ~703.04 billion RMB by the end of 2020. Dalian opened its first metro line in May 2003, making it the sixth city in mainland China with the operated metro system. As of December 2021, five lines have been put into operation with 201.03 km and 77 stations. The metro was widely used by commuters with an annual passenger volume of ~156 million passengers and an average daily passenger volume of about 426.6 thousand passengers. Due to the transportation needs of foreign tourists and residents, Dalian Metro plays a very important role in passenger transportation.

This study is to investigate the influence of the built environment on the walkability of a metro catchment area, focusing on the environmental perception and the objective supportiveness of the built environment. Therefore, the location and function of the metro station itself are used as the two bases for the classification of metro stations, so that the selected stations are more typical. Additionally, the central city is treated as our study area due to a large proportion of pedestrian connections and the dense distribution of metro lines. A large number of pedestrian flows can ensure the diversity of basic attributes such as social characteristics and the travel purposes of pedestrians. To effectively reflect the variability of the pedestrian environment of the catchment areas, eight stations, namely, Dalian Railway Station (DR-S), Harbor Square (H-S), South China Square (SC-S), Exhibition Center (E-C), Tsingniwa Bridge (TNW-B), Xi'an Road (XA-R), Friendly Square (F-S), and Zhongshan Square (ZS-S), were selected as the sampled stations. These stations can be divided into several types on the basis of their functions related to land use, including residential-dominated, commercial centers, transportation hubs, and landscape open types (Table 1).

**Table 1:** Overview of research sites.

**Location**	**Site name**	**Site type**	**Classification criteria and characteristics**	**Station lines**	**Number of entrances and exits**
Shahekou district	Xi'an road	Commercial center type	Located in the city center node; surrounded by commercial and entertainment offices; high flow of people and vehicles	Line 1/line 2	4
	Exhibition center	Open landscape type	Surrounded by famous attractions of the city; nearby buildings, roads, and environment are relatively well; significantly more people during holidays	Line 1	3
Xigang district	Dalian railway station	Transportation hub type	With subway interchange role; distribute various modes of transportation; high flow of people in the station;	Line 3/line 3 interval	2
Zhongshan district	Qingniwa bridge	Commercial center type	–	Line 2	3
	Friendship square	Commercial center type	–	Line 2	3
	Zhongshan square	Commercial center type	–	Line 2	6
	Harbor square	Open landscape type	–	Line 2	2
Ganjingzi district	South China square	Residence-led type	Surrounded by mainly residential areas; obvious residential group structure; commuter tide phenomenon	Line 1	3

**Figure 1:**
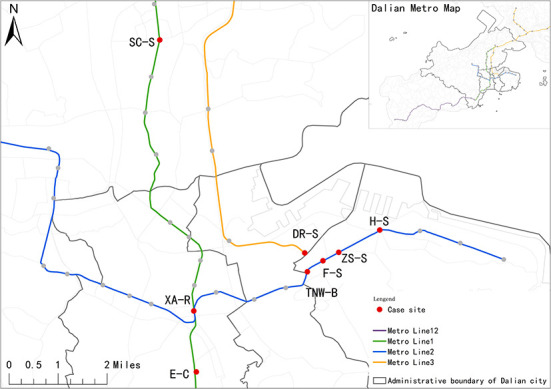
Case site location map.

In addition, previous studies on the walkability of the station usually use the buffer zone with 800-m radius as the catchment area of metro station (17, 81). Meanwhile, the *Chinese official Guidelines for Planning and Designing Areas along Urban Railways* specify that the rail impact area is the region that is about 500–800 m away from the station, within 15 min walking distance to the station entrance. Therefore, the area within 800 meters radius of the station (i.e., 10 min of walking time) is taken as the built environment background area for assessing walkability (Figure 1).

### Data

Two types of data were used in this study: (1) objective measurement of the built environment of the metro catchment area and (2) subjective perceptions of the walkability of the metro catchment area. Objective data such as pedestrian paths within the built environment were obtained through web-based big data, street maps, and field observations. The subjective perception data was collected by distributing a questionnaire to respondents and quantifying the results with the help of the five-scale Likert approach. The variables in the questionnaire were selected based on the relevant built environment factors affecting station walkability as mentioned above, with consideration of previously studied sidewalk assessment tools (74) and sidewalk levels of service (76). The questionnaire was initially tested among 10 graduate students and reviewed by experts in the field, followed by several modifications on the basis of feedback. In addition, 29 micro-scale built environment factors that promote or inhibit the walkability of the metro catchment area were selected, taking into account the distinctive characteristics of Dalian as a local tourist city. The factors were classified according to their characteristics and hierarchical structure into six design qualities: connectivity, convenience, safety, comfort, pleasure, and diversity (Table 2).

**Table 2:** Built environment variable attributes.

**Category**	**Variable**	**Description**	**Perspective**	**Data source**	**Expected direction**
Connectivity	Pedestrian network density	Total walkway length/station area	Objective	Road data	+
	Walkway area rate	Total walkway area/station area	Objective	Road data	+
	Walkway continuity	Mean score of respondents' evaluation of walkway continuity	Subjective	Survey questionnaire	+
	Intersection density	Number of intersections/station area	Objective	Field surveys/street maps	+
	Average block length	Sum of the average lengths of blocks/number of blocks	Objective	Field survey/street map	+
	End-of-road ratio	The total length of end streets/total length of pedestrian walkways	Objective	Road data	-
Convenience	Ease of pedestrian crossing	The average score of respondents' evaluation of the convenience of pedestrian crossing	Subjective	Survey questionnaire	+
	Ease of crossing at flyovers	The average score of respondents' evaluation of the convenience of crossing the street on pedestrian bridges	Subjective	Survey questionnaire	+
	Ease of crossing at underpasses	The average age score of respondents' evaluation of the convenience of crossing the street at underpasses	Subjective	Survey questionnaire	+
	Signage facilities	Average rating of respondents' evaluation of signage facilities	Subjective	Survey questionnaire	+
Security	Signal light facilities	Mean score of respondents' evaluation of signalization facilities	Subjective	Survey questionnaire	+
	Street lighting facilities	The average score of respondents' evaluation of street lighting facilities	Subjective	Survey questionnaire	+
	Degree in traffic safety	Average rating of respondents' evaluation of traffic safety	Subjective	Survey questionnaire	+
	Degree in social security	The average score of respondents' evaluation of the degree of social security	Subjective	Survey questionnaire	+
Comfort	Walkway width of the access	The average width of pedestrian walkways	Objective	Field survey/street map	+
	Quality of paving of footpaths	The average score of respondents' evaluation of pavement quality	Subjective	Survey questionnaire	+
	Sheltering facilities	The average score of respondents' evaluation of sheltering facilities	Subjective	Survey questionnaire	+
	Degree of obstruction	The average score of respondents' evaluation of the degree of obstruction	Subjective	Survey questionnaire	–
	Rest seating facilities around the walkway	The average score of respondents' evaluation of resting seating facilities around the walkway	Subjective	Survey questionnaire	+
Pleasure	Open space density	Area of public space and green space/station area	Objective	Remote sensing image	+
	Public artwork	The average score of respondents' evaluation of public artwork	Subjective	Survey questionnaire	+
	Degree of walkway cleanliness	The average score of respondents' evaluation of the cleanliness of the walkway	Subjective	Survey questionnaire	+
	Degree in greening and landscaping	The average score of respondents' evaluation of the degree of greenery and landscaping	Subjective	Survey Questionnaire	+
	Degree of pedestrian congestion	The average score of respondents' evaluation of crowdedness	Subjective	Survey questionnaire	–
Diversity	Density of living facilities	Number of living facilities/total area	Objective	Baidu POI	+
	Density of commercial facilities	Number of commercial facilities/total area	Objective	Baidu POI	+
	Density of recreational facilities	Number of recreational facilities/total area	Objective	Baidu POI	+
	Density of transportation facilities	Number of educational facilities/total area	Objective	Baidu POI	+
	Transparency of buildings on both sides of the walkway	The average score of respondents' evaluation of the transparency of buildings on both sides of the pedestrian walkway	Subjective	Survey questionnaire	+

### Survey

The questionnaire and field survey was conducted from August 2021 to March 2022. The field survey was divided into two parts: pre-survey and formal survey. The format, clarity, and wording of the content of the questionnaire were improved through a pre-survey of 30 respondents who randomly distributed the questionnaire. The content of the questionnaire was divided into three parts: basic personal information, travel characteristics, and pedestrian satisfaction with the built environment of the metro catchment area. Personal characteristics included information on gender, age, occupation, monthly income, and home address. The travel characteristics include the purpose of travel, the number of trips per week by metro, and the feeder modes of connecting to metro stations. The final part of the questionnaire collected respondents' subjective satisfaction with the built environment factors of the station area. During the formal research process, the familiarity of the metro catchment area was identified on the spot by asking respondents in advance if they frequently traveled to and from the area by metro. Thus, the questionnaires obtained for this study were representative of respondents who regularly take the metro for various purposes and more than two times per week. The researchers distributed a total of 867 questionnaires in clear weather on weekdays and weekends, respectively, with a valid sample size of 800 and an effective rate of 92.3%, of which 486 questionnaires were collected on weekdays, accounting for 60.8%.

According to the statistics of personal attributes in Table 3, the gender ratio of the respondents was ~50%. Teenagers and middle-aged people make up the majority of the total population. Nearly half of the respondents are commuters, followed by students. This shows the importance of the passengers who take the metro to work and school. Regarding income, most of the respondents are in the middle class, with 27.1% of the respondents in the 2,000–5,000 range and 30.8% in the 5,000–8,000 range. In addition, respondents take the metro about 4 times a week on average. 60.6% of the respondents choose walking as the mode to connect to the metro, which further demonstrates the validity of the sample. The purpose of travel is mainly shopping and dining, accounting for 39.9% of the total, followed by commuting to work, going home, and taking a leisurely walk, accounting for 29.1 and 20.8%, respectively. In summary, the survey sample has good representativeness in terms of both personal information and travel characteristics.

**Table 3:** Basic attributes statistics table.

**Attribute**	**Subgroup**	**Numerical value**
Gender	Male	51.4%
	Female	48.6%
Age	18–25 years old	30.1%
	26–35 years old	27.1%
	36–45 years old	20.2%
	46–55 years old	10.5%
	>56 years old	12.1%
Occupation	Students	22.5%
	Commuters	49.0%
	Freelance	11.4%
	Retired	10.6%
	Government employee	0.4%
	Others	6.1%
Income	<2,000	23.5%
	2,000–5,000	27.1%
	5,000–8,000	30.8%
	8,000–10,000	13.0%
	>10,000	5.6%
The average number of subway rides per week		4
Purpose of travel	Going to and from work, going home	29.1%
	Going to and from school	7.9%
	Shopping and eating	39.9%
	Leisurely walk	20.8%
	Other	2.3%
Connection method	Walking	60.6%
	Bicycle	2.8%
	Bus	23.3%
	Taxi, dropshipping	9.2%
	Electric car	0.9%
	Private car	3.2%

## Methodology

A total of 867 valid questionnaires were generated during the research. Based on the available data collected, this study designs a machine learning-based prediction algorithm to assign weight to the variables. The algorithm consists of two modules: principal component analysis (PCA) and feedforward neural network.

Among them, PCA can effectively reduce the dimensionality of different types of data while maintaining multiple variations in data samples. It helps to eliminate the multicollinearity that destroys the statistical significance of independent variables in the data set, thus making the statistical results more accurate. At the same time, the value relationships among multiple data are explained more clearly by assigning variable weight labels. We use PCA to reduce the training time and avoid overfitting problems. When implementing PCA, our model's input is the original data, PCA transforms the data to a new subspace. Then we feed the output to the feedforward neural network. We designed the feedforward neural network with four fully-connected layers with 29, 128, 64, and 29 nodes in each layer. The feedforward neural network learns the relationship between the input and output manually. We use Adam Optimizer to train the feedforward model because Adam can tune the learning rates automatically. To compare the difference between the prediction and groundtruth. We leverage the MSE loss function. To get a stable neural network model, we train our model for 250 epochs. We can see from **Figure 3**, the model is stable when it is trained more than 50 epochs. The machine learning-based variable weight prediction algorithm effectively combines the objective and subjective to eliminate the scale effect and assign more objective weight to the variables. Based on the collected data, the correlation between different kinds of variables is explored with the help of PCA, the feedforward neural network is utilized to learn the weights variation of each variable with the guiding of the weights generated by PCA. Compared with the traditional correlation analysis and regression analysis, it effectively enhances the robustness of weight prediction and makes the research results of weights have the obvious advantages of being more objective and having higher credibility.

**Figure 2:**
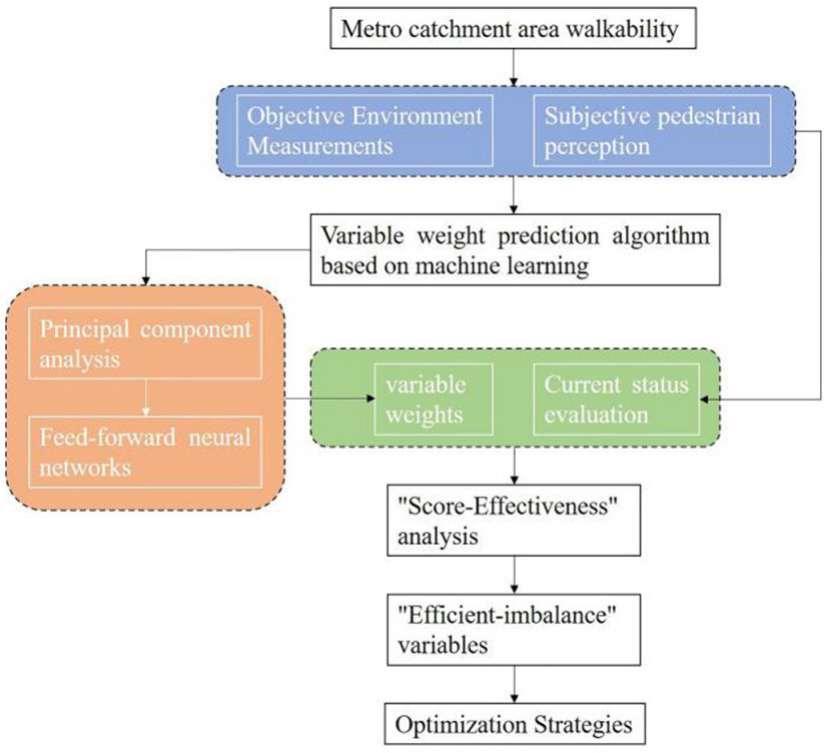
Research framework.

The weight of the variable represents the degree of the effect of the pedestrian environment factor on the walkability of the metro catchment area, and some variables even have a critical role. It is reasonable to discover the current problems and directions for improvement in the pedestrian environment by deeply exploring the individual factors that significantly affect the walkability of the metro catchment area. Therefore, this study combines the weighting results with the relevant built environment evaluation scores of the catchment area's current walkability to construct a “Score- Effectiveness” (S-E) two-dimensional quadrant diagram. This framework allows for a comprehensive analysis of the two-way correlation between the walkability performance of each built environment variable in the catchment area and its corresponding weight. This can fully illustrate the correspondence between the current status scores of variables and their effectiveness, to dig out the most sensitive existing problems related to walkability in the catchment area. In particular, for those built environment factors with low scores and high effectiveness, targeted improvement strategies that minimize the cost and maximize the benefit can significantly improve the walkability of the station catchment area. These strategies can precisely and effectively enhance the objective walkability and the subjective pedestrian friendliness of the station catchment area (Figure 2).

Due to the difference in the unit of selected data, the variables need to be normalized before entering the algorithm model. The evaluation results of the subjective variables are obtained by averaging the five-scale Likert scores of the eight stations, which ranged between 1 and 5 scores. For the objective variables, the scores vary in magnitude due to the existence of different scales. Therefore, the mean of the objective variables was normalized, followed by transferring into the same interval as the subjective scores, thus eliminating the problem of subjective and objective scales, as follows.


$$\begin{array}{l} \text{Y}={\text{y}}_{\text{MIN}}+\frac{{\text{y}}_{\text{MAX}}-{\text{y}}_{\text{MIN}}}{{\text{x}}_{\text{MAX}}-{\text{x}}_{\text{M}I\text{N}}}\times \left(\bar{\text{x}}-{\text{x}}_{\text{MIN}}\right) \end{array}$$

Where *Y* is the normalized mapped value; *y*_*MAX*_ is the maximum value of the mapped target interval; *y*_*MIN*_ is the minimum value of the mapped target interval; *X*_*MAX*_ is the maximum value in the original dataset; *X*_*MIN*_ is the minimum value in the original dataset; $\bar{x}$ is the average value in the original dataset.

## Results

### Subjective and objective variable scores

The scores of each variable are shown in Table 4. Friendly Square (F-S) has 8 variables which ranked first (8), including the rate of walkway area, social security, walkway width, shade facilities, obstacle barrier, resting seat facilities around the walkway, and pedestrian congestion. By contrast, the Zhongshan Square (ZS-S) metro catchment area received 8 variables with the lowest value. It is possible that F-S is located in the financial and commercial center of Renmin Road, the busiest area in Dalian, and serves as an important node connecting transportation hubs and commercial areas such as Dalian Railway Station, Shengli Square, and Tianjin Street. Because of the strong pedestrian demand itself, the construction of pedestrian paths and various services in the catchment area provide pedestrians with high-quality, experiential walking spaces. Moreover, the characteristics of high pedestrian flow also make the two negative variables of obstacle barrier degree and pedestrian crowding degree score significantly larger than other stations. The ZS-S catchment area is one of the most famous squares in Dalian attributed to the circular square of ~168 meters in diameter. At the beginning of its planning and construction, a long and fatiguing walking path for pedestrians was designed. In addition, as a famous and historically significant square in Dalian, ten avenues radiate out from the area, which results in complex road conditions, high traffic flow, and lack of signalization. As a consequence, it causes many concerns about pedestrian safety.

**Table 4:** Evaluation indicator scores for each station area.

**Indicator layer**	**Dalian Railway Station (DR-S)**	**Harbor Square (H-S)**	**South China Square (SC-S)**	**Exhibition Center (E-C)**	**Tsingniwa Bridge (TNW-B)**	**Xi'an Road (XA-R)**	**Friendly Square (F-S)**	**Zhongshan Square (ZS-S)**	**Objective interval value/subjective average**
Pedestrian network density (a1)	35.66	27.91	26.01	33.52	37.43	37.92	37.65	34.07	3.61
Walkway area rate (a2)	16.10%	10.77%	12.98%	14.02%	17.35%	14.80%	17.88%	15.34%	3.33
Walkway continuity (a3)	4.03	3.86	4.00	3.55	3.86	3.38	3.79	3.72	3.78
Intersection density (a4)	51.23	35.81	12.43	51.23	64.66	45.76	89.71	90.52	3.19
Average block length (a5)	208.59	199.54	244.76	210.89	206.68	192.74	166.76	165.24	2.72
End-of-road ratio (a6)	2.32%	1.19%	1.64%	3.27%	2.13%	2.50%	2.25%	1.15%	2.71
Ease of pedestrian crossing (a7)	4.00	3.86	3.90	3.48	3.69	3.62	3.38	3.21	3.64
Ease of crossing at flyovers (a8)	4.00	3.18	3.47	3.29	3.14	3.18	3.18	2.00	3.18
Ease of crossing at underpasses (a9)	4.23	3.72	3.70	3.26	3.52	3.57	2.90	3.69	3.57
Signage facilities (a10)	4.00	4.03	4.10	3.26	3.79	3.86	3.48	3.83	3.79
Signal light facilities (a11)	4.00	3.59	4.10	4.03	4.17	4.17	4.00	3.59	3.96
Street lighting facilities (a12)	4.10	4.03	4.27	4.13	3.97	4.28	4.31	3.97	4.13
Degree of traffic safety (a13)	3.93	3.69	3.93	3.39	4.00	3.90	3.83	3.76	3.80
Degree of social security (a14)	4.20	4.10	4.37	4.42	4.31	4.14	4.48	3.97	4.25
Walkway width of the access (a15)	4.51	3.86	3.87	4.18	4.66	3.92	4.75	4.50	4.28
Quality of paving of footpaths (a16)	3.97	3.59	3.97	4.06	3.59	4.07	3.86	3.45	3.82
Sheltering facilities (a17)	3.63	3.59	3.40	2.84	3.14	2.38	3.86	3.48	3.29
Degree of obstruction (a18)	3.63	3.21	3.70	2.84	3.55	2.48	4.17	3.55	3.39
Resting seating facilities around the walkway (a19)	3.77	2.62	3.23	2.55	3.24	2.45	3.90	3.72	3.18
Open space density (a20)	2.69%	3.83%	5.71%	3.75%	3.60%	4.72%	4.71%	13.11%	1.99
Public artwork (a21)	4.03	4.10	3.53	4.06	3.24	2.90	3.86	4.17	3.74
Degree of walkway cleanliness (a22)	4.13	4.07	4.03	4.23	3.59	4.03	3.86	4.17	4.01
Degree of greenery and landscape (a23)	3.83	4.14	3.80	3.94	3.17	3.38	4.17	4.24	3.83
Degree of pedestrian congestion (a24)	3.67	3.52	3.60	3.48	3.66	3.10	3.90	3.72	3.58
Density of living facilities (a25)	95.49	77.59	129.31	46.25	153.68	253.65	206.90	239.73	3.01
Density of commercial facilities (a26)	188.50	126.83	268.57	283.00	250.17	346.16	262.11	271.06	3.24
Density of recreational facilities (a27)	3.98	3.98	4.48	28.35	8.46	23.38	6.47	6.47	2.10
Density of transportation facilities (a28)	14.92	5.47	7.96	3.98	14.42	8.95	14.92	13.43	3.39
Transparency of buildings on both sides of the walkway (a29)	3.90	3.31	3.80	3.23	4.03	4.38	4.03	3.79	3.81

As for the subjective variables, the width of the pedestrian paths in the catchment area, the degree of social security, street lighting facilities, and the neatness of the pedestrian paths were generally recognized by the respondents; the scores of the rest seating facilities, shading facilities and the degree of barrier blockage around the pedestrian paths were significantly lower than the other variables. Combined with the field survey and interviews, most walking paths in the catchment area have problems such as a lack of service facilities, space encroachment, and unclear management.

### Machine learning to determine variable weights

**Figure 3:**
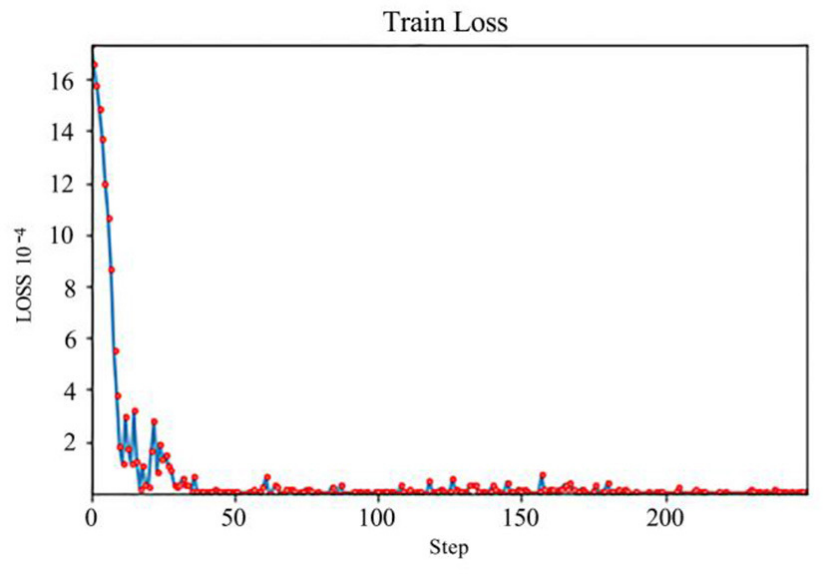
Convergence process of loss function of feedforward neural network.

For the designed algorithm, the PCA is used to calculate the weights of each variable based on the collected data, the output of the weight calculated by the PCA is used as the data labels, and the feedforward neural network is used to learn the robust mapping from the variable samples to the weight labels. The loss function of this feedforward neural network gradually decreases during the training process until the network converges. The convergence process is shown in Figure 3, where the horizontal coordinate is the number of data iterations and the vertical coordinate is the loss value.

**Figure 4:**
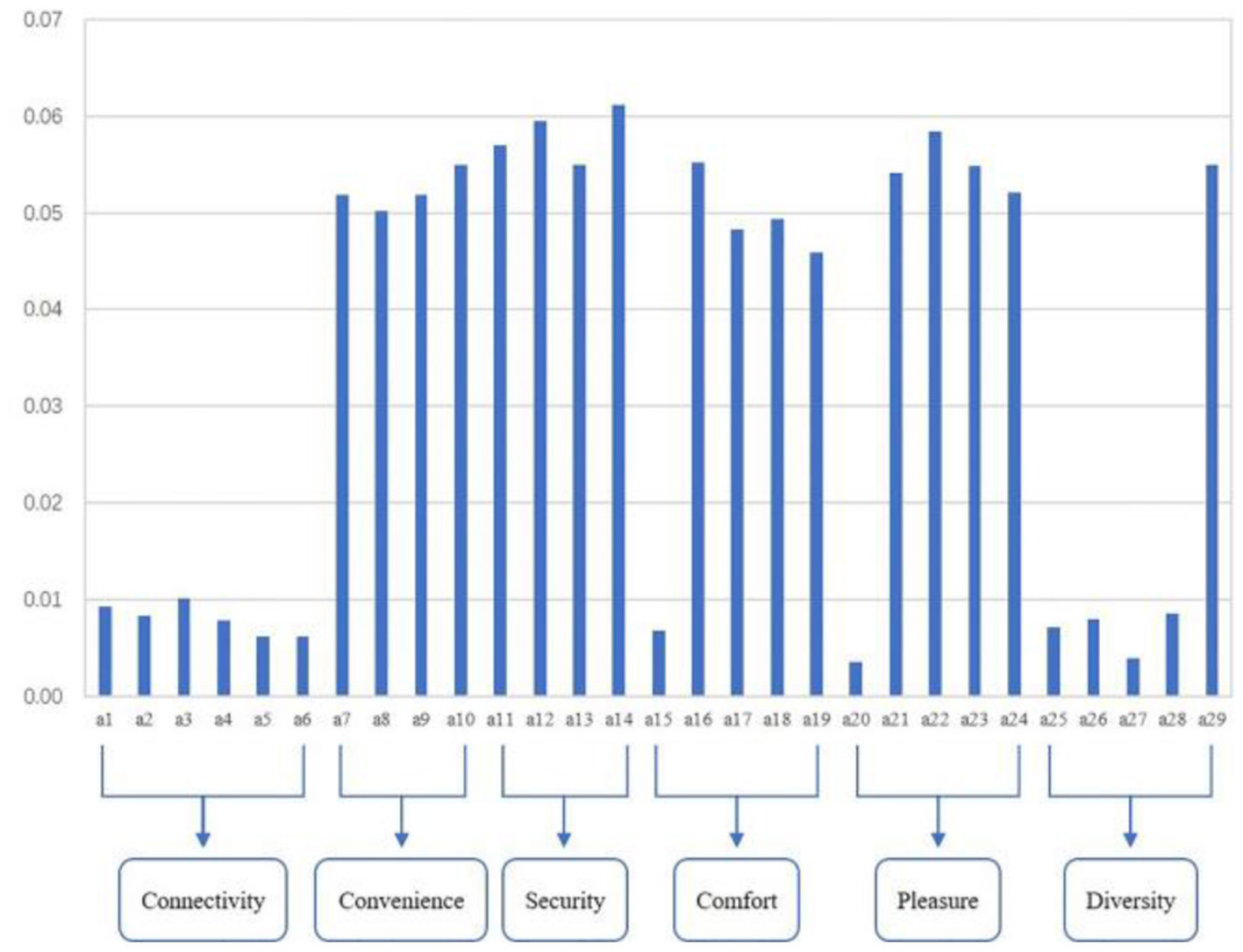
Distribution of variable weights.

The modeling results of variable weights are shown in Figure 4. Factors related to walking connectively, include a1 (Pedestrian network density), a2 (Walkway area rate), a3 (Walkway Continuity), a4 (Intersection density), a5 (Average block length), and a6 (End-of-road ratio), are <0.01.

As a comparison, convenience and safety variables such as a7 (Ease of pedestrian crossing), a8 (Ease of crossing at flyovers), a9 (Ease of crossing at underpasses), a10 (Signage facilities), a11 (Signal light facilities), a12 (Street lighting facilities), a13 (Degree of traffic safety) and a14 (Degree of social security) have weights above 0.05, indicating the important role on affecting the walkability of metro areas. Among these convenience and safety factors, metro users may have many concerns about social security (a14 with the highest weight of 0.062) when connecting the metro transit.

Regarding the comfort and pleasure variables, only a15 (Walkway width of the access) and a20 (Open space density) have weights below 0.01, while the remaining factors have significantly greater weights than the previous two, including a16 (Quality of paving of footpaths), a17 (Sheltering facilities), a18 (Degree of obstruction), a19 (Resting seating facilities around the walkway), a21 (Public artwork), a22 (Degree of cleanliness of the walkway), a23 (Degree of greenery and landscape), and a24 (Degree of pedestrian congestion). Within the comfort and pleasantness variables, people may place more importance on the cleanliness of the walkway (a12 with the highest weight of 0.058).

On the contrary, most of the variables in diversity have weights below 0.01, such as a25 (Density of living facilities), a26 (Density of commercial facilities), a27 (Density of recreational facilities), and a28 (Density of transportation facilities). And the weight of a29 (Transparency of buildings on both sides of the walkway) is higher than 0.05, which is five times more than the weight values of other variables in the diversity. This indicates that metro users may care more about the façade form of buildings on both sides of the walkway than the density of various service facilities in the catchment area.

### “Score-effectiveness” suitability analysis

In this study, we construct a framework of the “*Score- Effectiveness*” fit quadrant in which the horizontal coordinates are the weights obtained from the variable weight prediction algorithm and the vertical coordinates are the scores of the current variables. The baseline of the four quadrants is the mean value of the weights and satisfaction scores as the parallel axes of the horizontal and vertical coordinates, respectively. To form an effective, precise, and efficient optimization strategy based on field surveys and research.

**Figure 5:**
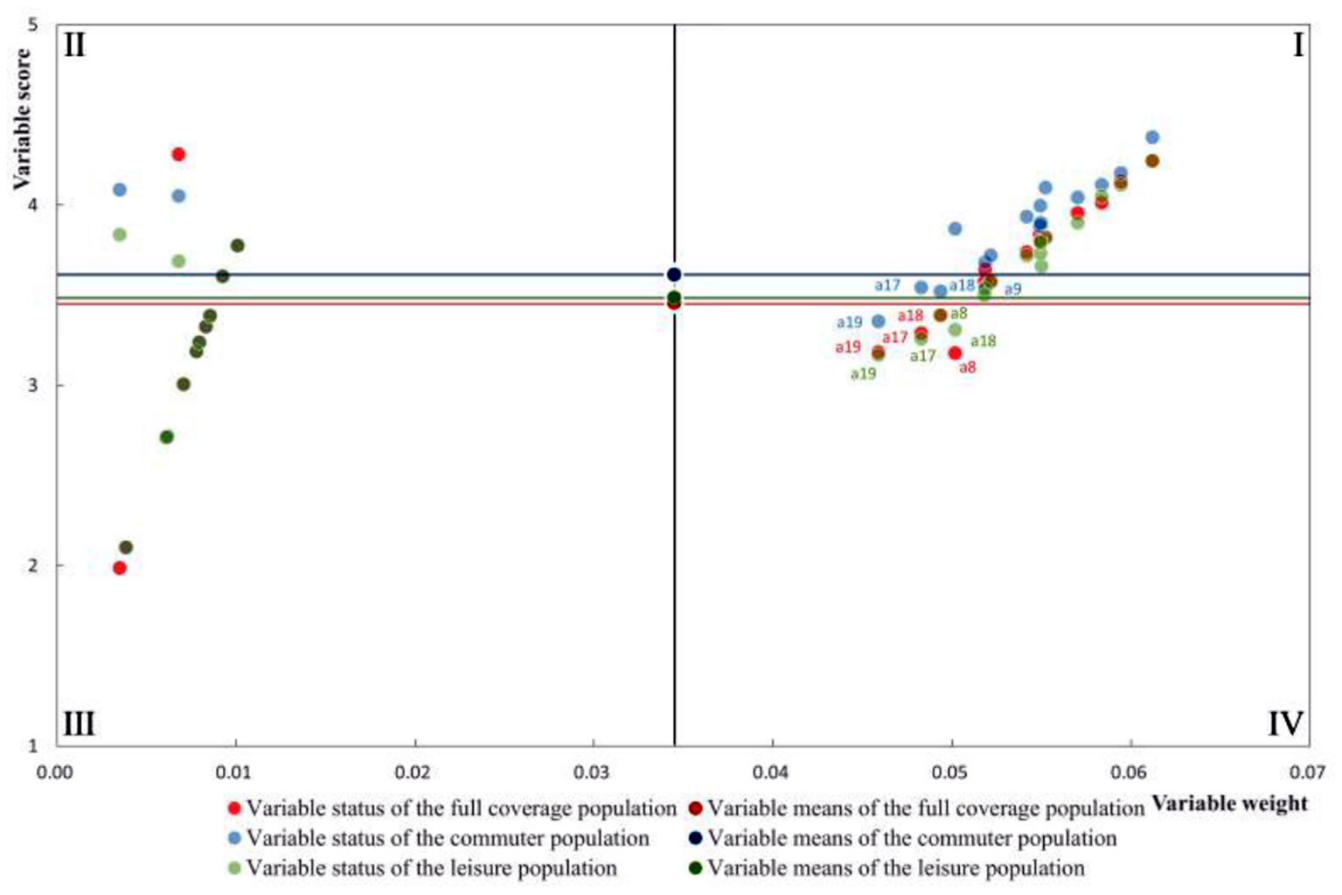
“Score-effectiveness” suitability quadrant.

As shown in Figure 5, both the weights and satisfaction results of variables in quadrant I are higher, indicating that pedestrians are more satisfied with the current situation of this variable, which belongs to the efficient and balanced type. The variables in quadrant II are low-efficiency and high-quality, meaning that pedestrians are also satisfied with these low-efficiency variables and only need to maintain the status quo for these aspects. The variables in quadrants III and IV are the inefficient and poor quality type and the efficient and imbalanced type, respectively. The variables in quadrant III have slightly less influence on satisfaction, and appropriate optimization can be performed at appropriate time points for the weak points with low ratings. Variables in quadrant IV are characterized by high performance and low scores, which have a greater impact on satisfaction while scoring low. This indicates that the efficient imbalance-type variables are built at a relatively low level, but have a direct and important impact on the friendliness of supporting pedestrian walking. Therefore, the relevant variables need to be improved and optimized urgently to be able to improve the current level of metro catchment area walkability in a targeted and maximally effective way.

By counting the data from the questionnaire, as shown in Table 3 above, the respondents in the metro catchment area can be divided into commuting and leisure categories based on different travel purposes. Among them, 37% are commuters (commuting to and from work and school) and 60.7% are leisure people (leisurely walking, shopping and dining). Using the “Score-Effectiveness” model, the efficient imbalance-type variables under the three categories of full coverage, commuting, and leisure were identified by stratifying the purpose of the population trips, respectively. As shown in Figure 5, the efficient imbalance-type variables are consistent for full coverage and leisure populations, including a8 (Ease of crossing at flyovers), a17 (Sheltering facilities), a18 (Degree of obstruction), and a19 (Resting seating facilities around the walkway). For the commuter population, a9 (Ease of crossing at underpasses), a17, a18, and a19 are in low rating, high performance status. In general, a17, a18, and a19 are fixed efficient imbalance variables. While comparing the commuting and leisure population, the former cares more about the performance of a9 and the latter is focused on a8. Both a8 and a9 are crossing facilities, which also shows that the two types of people, commuters and leisure, have different needs for station domain walkability and have differential judgments on crossing forms. The flyover plays the role of outdoor landscape while assuming the traffic function. The leisure population prefers open crossing space, while the commuter population finds the underpass crossing more convenient.

## Discussion

In this study, both subjective and objective perspectives are applied to evaluate the walkability of the metro catchment area. An approach of predicting the weight of variables based on machine learning technique is developed to accurately measure the importance of built environment factors that affect walkability. Among the selected built environment factors, the shade facilities, obstacle barriers, and resting seats around the pedestrian paths were found to be highly effective and imbalanced variables recognized as fixed by the population. indicating the necessity for improvement. Meanwhile, the ease of crossing the flyovers and underpasses are additional efficient imbalance-type variables for the leisure and commuting populations. On the basis of the analysis in terms of the five efficient imbalance-type variables, this paper proposes targeted optimization strategies accordingly.

First, pedestrian bridges, an important measure to improve the convenience of walking, are suggested to be installed at traffic nodes such as main roads with high traffic flow and large safety hazards. In Dalian city, motorways, tramways, sidewalks, etc. are crisscrossed in the metro catchment area, causing major hidden dangers to the convenience and safety of pedestrians crossing the street. However, our field observation found that overpass crossing facilities in Dalian are extremely absent, which makes walkers inconvenient for crossing the road. Therefore, overpass crossing facilities should be added at appropriate locations around the catchment area to enhance walkability and form a friendly walking space to separate pedestrians and vehicles (76). In addition, the design of the overpass crossing should take care of the use of vulnerable groups such as the elderly. With the help of barrier-free designs (e.g., elevators and ramps), an effective and friendly pedestrian corridor is formed to eliminate the pedestrian's psychological barriers to walking caused by excessive slopes.

Second, during the interviews, most of the interviewees reported that the interior of the underpass was too dark, poorly tidied, and had a low level of security. It is recommended that large glass areas be used at the entrances and exits of underground passages to introduce a greater range of natural light. Strengthen the relevant management personnel to improve efforts, pay attention to health cleaning, post-maintenance and social security. Reduce the situation of mobile vendors occupying underground traffic sex paths and adopt time-sharing and centralized management. In addition, the underground passageway of the metro catchment area can and should be combined with the surrounding underground commercial space to maximize the use of the pedestrian flow and vitality brought by the station. The benefits are maximized while reducing the pressure of pedestrian flow in the ground commercial walkway.

Third, it is suggested to appropriately improve the ability of the shading facilities to cope with extreme weather. Possible measures include planting street trees along the avenues, building eaves, and setting corridors and canopies. According to the field study, there are very few sheltering facilities along the route to the metro stations, which makes metro users uncomfortable walking to the stations in bad weather. It is suggested to use some flexible sheltering facilities, such as folding devices similar to umbrellas on both sides of the walkway. In addition, shelter facilities can be also designed by integrating the functions of greening and rainwater collection collectively. These techniques will not only improve the overall environmental quality but also makes resources to be utilized more effectively.

Fourth, enhancing the environmental management and optimization in the metro catchment area is encouraged. The field survey also found that the pedestrian space in the catchment area is seriously encroached upon by commercial and advertising obstacles. Excessive space encroachment can directly lead to a significant reduction in walkability because of the increasing traffic chaos and safety risk. Therefore, it is recommended that the relevant authorities should develop management measures to prohibit commercial and advertising barriers in the pedestrian space, particularly around the entrance of metro stations where passengers are usually crowded, to reduce its negative impact on walkability.

Finally, the metro catchment area should be provided with public seating and resting space approximately. In this study, we found that resting seating facilities along the walkway have a positive impact on station area walkability, which is in line with the results of previous studies (77, 82). Dalian is a city with many open squares, and most of the metro catchment areas are accompanied by city squares spatially. However, squares with large sizes may cause fatigue to pedestrians and visitors, and that is why approximate allocation of resting facilities is particularly important to improve the willingness of walking. Therefore, increasing the number of resting seats along the pedestrian walkway can not only make walking less tiring but also improve the neighborhood vitality of the station area and enhance the attractiveness of the city.

## Conclusion

Currently, the urban metro system in China is developing rapidly. However, the development of the metro catchment areas, particularly the planning and management of walking-related facilities, does not meet well with demands (i.e., comfort, convenience, and safety) of metro passengers. Considering that the land use has been planned, the optimization of the built environment of the metro catchment area is easier to implement and more effective than the reorganization of built environment factors. In this paper, subjective and objective variables of metro catchment area walkability are effectively combined. Through the innovative machine learning-based variable weight prediction algorithm, the weight value of each variable is obtained more scientifically and objectively. Based on this, this study further constructs a “Score-Effectiveness” suitability system to precisely identify the built environment factors that should be improved to enhance walkability in metro catchment areas.

The results of the study help to understand the priorities of built environment factors that affect the walkability of metro catchment areas, as well as to pinpoint the existing problems related to the walking connection to metro stations. A comparative analysis of the stratification of the population was conducted to find out the differentiated demand for metro catchment area walkability by populations who travel for both commuting and leisure purposes. This study provides relevant governments and planners with targeted and effective optimization strategies to make the metro areas more walkable. The major contributions of this study include: (1) to construct the “Score-Effectiveness” framework to identify the imbalanced factors in terms of the built environment, and hence, contribute to providing targeted policies to improve the walkability of metro areas; (2) to collectively identify the weight of objective and subjective built environment factors through developing a machine learning-based approach, which makes it possible to compare the importance of objective and subjective built environment factors.

However, there are limitations to this study. First, due to the difficulties in data collection, some built environment variables such as traffic congestion, noise, and air pollution (83) that may potentially affect walkability are unselected approximately. Further, we just selected eight sampled metro stations for analysis, which may cause some bias potentially. Future studies can expand the sample city size to enhance comparability.

## Data availability statement

The original contributions presented in the study are included in the article/supplementary material, further inquiries can be directed to the corresponding author/s.

## Author contributions

WX and YG: conceptualization. PZ and YG: funding acquisition, validation, and writing—review and editing. WX: formal analysis, supervision, methodology, and writing—original draft. BL and LS: investigation. MX: machine learning algorithm design and practice. All authors contributed to the article and approved the submitted version.

## Funding

This study was supported by the National Natural Science Foundation of China (No. 51978447) and the Major Training Project of Team+Project in Tianjin (No. XC202061).

## Conflict of interest

The authors declare that the research was conducted in the absence of any commercial or financial relationships that could be construed as a potential conflict of interest.

## Publisher's note

All claims expressed in this article are solely those of the authors and do not necessarily represent those of their affiliated organizations, or those of the publisher, the editors and the reviewers. Any product that may be evaluated in this article, or claim that may be made by its manufacturer, is not guaranteed or endorsed by the publisher.
